# Mesenchymal stem cells: A comprehensive methods for odontoblastic induction

**DOI:** 10.1186/s12575-021-00155-7

**Published:** 2021-09-15

**Authors:** Benson Koh, Nadiah Sulaiman, Sharifah Nursyazwani Shahirah Wan Ismadi, Roszalina Ramli, Siti Salmiah Mohd Yunus, Ruszymah Bt Hj Idrus, Shahrul Hisham Zainal Ariffin, Rohaya Megat Abdul Wahab, Muhammad Dain Yazid

**Affiliations:** 1 Centre for Tissue Engineering & Regenerative Medicine, Faculty of Medicine, Universiti Kebangsaan Malaysia Medical Centre, Jalan Yaacob Latif, 56000 Cheras, Kuala Lumpur Malaysia; 2 Department of Oral & Maxillofacial Surgery, Faculty of Dentistry, Universiti Kebangsaan Malaysia, Jalan Raja Muda Abdul Aziz, 50300 Kuala Lumpur, Malaysia; 3 Department of Biological Sciences and Biotechnology, Faculty of Science and Technology, Universiti Kebangsaan Malaysia, 43600 Bangi, Selangor Malaysia; 4 Department of Orthodontic, Faculty of Dentistry, Universiti Kebangsaan Malaysia, Jalan Raja Muda Abdul Aziz, 50300 Kuala Lumpur, Malaysia

**Keywords:** Mesenchymal stem cells, Dental cells, Odontoblastic differentiation, Dentinogenesis

## Abstract

**Background:**

In the area of oral and maxillofacial surgery, regenerative endodontics aims to present alternative options to conventional treatment strategies. With continuous advances in regenerative medicine, the source of cells used for pulp tissue regeneration is not only limited to mesenchymal stem cells as the non-mesenchymal stem cells have shown capabilities too. In this review, we are systematically assessing the recent findings on odontoblastic differentiation induction with scaffold and non-scaffold approaches.

**Methods:**

A comprehensive search was conducted in Pubmed, and Scopus, and relevant studies published between 2015 and 2020 were selected following the PRISMA guideline. The main inclusion criteria were that articles must be revolving on method for osteoblast differentiation in vitro study. Therefore, in vivo and human or animal clinical studies were excluded. The search outcomes identified all articles containing the word “odontoblast”, “differentiation”, and “mesenchymal stem cell”.

**Results:**

The literature search identified 99 related studies, but only 11 articles met the inclusion criteria. These include 5 odontoblastic differentiation induction with scaffold, 6 inductions without scaffolds. The data collected were characterised into two main categories: type of cells undergo odontoblastic differentiation, and odontoblastic differentiation techniques using scaffolds or non-scaffold.

**Conclusion:**

Based on the data analysis, the scaffold-based odontoblastic induction method seems to be a better option compared to the non-scaffold method. In addition of that, the combination of growth factors in scaffold-based methods could possibly enhance the differentiation. Thus, further detailed studies are still required to understand the mechanism and the way to enhance odontoblastic differentiation.

## Introduction

Regenerative endodontics (RE) is a new division of tissue engineering and regenerative medicine. In the area of oral and maxillofacial surgery, it aims to present alternative options to conventional treatment strategies. The objective in RE is to reconstruct maxillofacial defect and also to replace the dying pulp with scaffolds, healing promoting factors, and cell therapies with the aim of regenerating new pulp and dentine within the root canal system [1]. Advances in regenerative medicine and tissue engineering along with the introduction of new sources of stem cells have led to the possibility of pulp tissue regeneration. To translate this effectively to the clinic setting, pre-clinical and clinical studies are ongoingly conducted in order to introduce the most effective, efficient and promising treatment for dental field.

Dental stem cells (DSCs) are multipotent cells with high proliferative capacity that can differentiate into multiple cell lineages. The different types of mesenchymal stem cell (MSC) population isolated from dental tissues include dental pulp stem cells (DPSCs), stem cells from human exfoliated deciduous teeth (SHED), stem cells from apical papilla (SCAP), dental follicle stem cells (DFSCs), gingival mesenchymal stem cells (GMSCS) and periodontal ligament stem cells (PDLSCs). MSC show CD10, CD13, CD44, CD73, CD105 phenotype but do not express CD31 or CD45. These stem cells are evolving as a promising alternative treatment for various tissue defects due to the less invasive procedure of isolation and high proliferation rate compared to bone marrow aspiration.

**Fig. 1:**
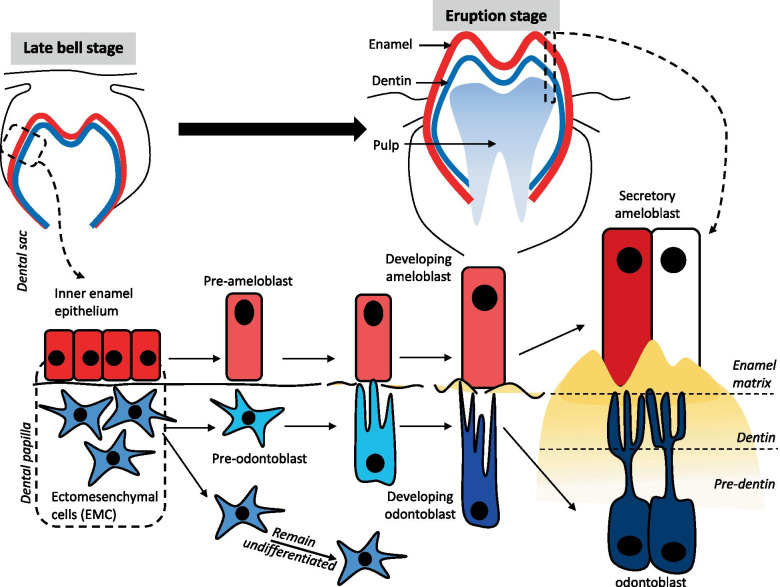
The dentinogenesis starts at the late bell stage. The inner dental epithelium induced by various signalling molecules such as Wnt, Runx-2, and TGF-β differentiate into pre-ameloblast and subsequently become secretory ameloblast. Whilst, the ectomesenvhymal cells reside in dental papilla becomes the pre-odontoblast and the other cells remain undifferentiated. Pre-odontoblast develop into tall and polarized with the nucleus away from the dental membrane known as Tomes fibers. Tomes fibers connect with the cells at surface of dentin and continuously secrete organic matrix which composed of proteoglycans, glycoproteins, and collagens. The matrix accumulates as unmineralized layer (pre-dentin) and gradually mineralizes to form dentin

Dentinogenesis is a dentin formation by odontoblasts that differentiate from ectomesenchymal cells (EMC) of dental papilla located at the periphery of the dental pulp that continues throughout the life of a tooth. It is initiated by the inductive influence of the undifferentiated cells of the inner enamel epithelium, involving molecular signaling pathways, such as Wnt, Runx-2, and TGF-β. As soon as the cells of the inner enamel epithelium differentiates into pre-ameloblasts, the underlying cells of the dental papilla will stop dividing and will form two daughter cells. Out of these two daughter cells, one of them will differentiate into pre-odontoblasts, while the other one will remain undifferentiated in the pulp of a tooth which can be activated any time by an external stimulus. The newly differentiated odontoblasts will lay down the dentinal matrix at the end of its cytoplasmic extensions. This first layer of unmineralized dentinal matrix is called the mantle pre-dentin. The word pre-dentin refers to the unmineralized dentinal matrix. As soon as the pre-dentin mineralizes, it will become the mature dentin. This process is also summarized in Fig. 1.

Primary and secondary dentinogenesis, occurring before and after an eruption, respectively, are physiologic processes, whereas tertiary dentinogenesis, which can be either reactionary or reparative, occurs in response to injury. Primary and secondary dentins are histologically similar and are deposited at 4 and 0.4 µm/d, respectively [2]. Reactionary dentinogenesis is the secretion of a tertiary dentine matrix by surviving odontoblasts in response to an appropriate stimulus. The dentin matrix is permeable by its tubular structure, and, therefore, after an injury to the tooth and/or subsequent restorative procedures, this may allow molecules to diffuse and contact the pulp. Such substances may include bacteria, toxins, and/or dentin matrix proteins (DMPs). Because the pulp is enclosed by a rigid, mineralized tissue shell, dentin matrix degradation by acid, bacterial products begins before the disease process reaches the pulp.

Notably, growth factors derived from the dentin have been shown to reach and stimulate the odontoblast layer, inducing new dentine secretion in those areas of the dentin-pulp complex that are in direct tubular connection with the traumatic agent. Culturing stem cells with different vehicles can induce them to differentiate into specific target tissues. For example, dexamethasone and ascorbic acid in culture media are widely used to induce osteogenic differentiation [3]. Tooth germ cell-conditioned medium (TGC-CM) has been introduced for its inductive potential in odontoblastic differentiation [4], which, in this study, TGC-CM is prepared from rats in two different stages, embryonic and neonatal, and they cultured dermal multipotent stem cells in these two media. It is observed that embryonic TGC-CM was more bone inductive rather than odontoblastic. Wang et al. [5] reported that culturing DPSCs using porcine-derived TGC-CM resulted in a better regulated odontoblast-like cell layer formation compared with human-derived TGC-CM.

In this review, we are focusing on the recent findings on various methods to induce odontoblastic differentiation. It is categorized by scaffold and non-scaffold approaches.

## Methods

### Review question

This review was undertaken to determine the variety of methods to induce the differentiation of cells to odontoblast. Different types of mesenchymal stem cells and non-mesenchymal stem cells are used in the selected studies. This review was conducted in accordance to the Preferred Reporting Items for Systematic Reviews and Meta-Analyses (PRISMA) guidelines.

### Search strategy

Using pre-specified inclusion and exclusion criteria, we identified all English publications reporting the odontoblastic differentiation in vitro by searching two electronic databases which are PubMed and Scopus. The query was specified from 2015 to 2020 using the following search terms: odontoblast* AND differentiation AND mesenchymal stem cell.

### Selection criteria

The year limit for searches was from 2015 to 2020, and only studies published in English were considered. The search outcomes identified all articles containing the word “odontoblast”, “differentiation”, and “mesenchymal stem cell”. Databases were searched individually to ensure all relevant studies were considered. The titles and abstract were carefully screened for eligibility related to the topic of interest. In this review, we were focusing on in vitro study revolving on method used to differentiate the cell, effectively. Therefore, in vivo, and human/animal clinical studies were excluded. Review articles, news articles, letters, editorials, and case studies were excluded from the search.

### Data extraction and management

Data were extracted from each eligible article by three reviewers working independently. The selected papers were screened in several phases prior to inclusion. First, titles that were not relevant to the topic were excluded. Next, abstracts of the papers were screened, and unrelated studies were excluded. All duplicates were removed. The following data were sought into 2 different categories, i.e., odontoblastic induction using scaffold or non-scaffold. The type of cell and method for odontoblastic induction were included in each category and were summarized from the selected studies: authors, year, cell type, odontoblastic induction methods, results, and conclusion.

### Risk bias assessment

The three independent reviewers evaluated the risk of bias of the included studies using an adapted version of the Office of Health Assessment and Translation (OHAT) risk of bias tool [6]. This tool of assessment includes the risk of bias in the following domains: (1) selection bias; (2) performance bias; (3) detection bias; (4) attrition bias; (5) reporting bias. Studies were judged as having a low risk of bias ( +), high risk of bias (-), unclear risk of bias (?), and not applicable (NA). Any disagreement on the risk of bias assessment was resolved by further discussion between the reviewers.

## Results

### Search results

**Fig. 2:**
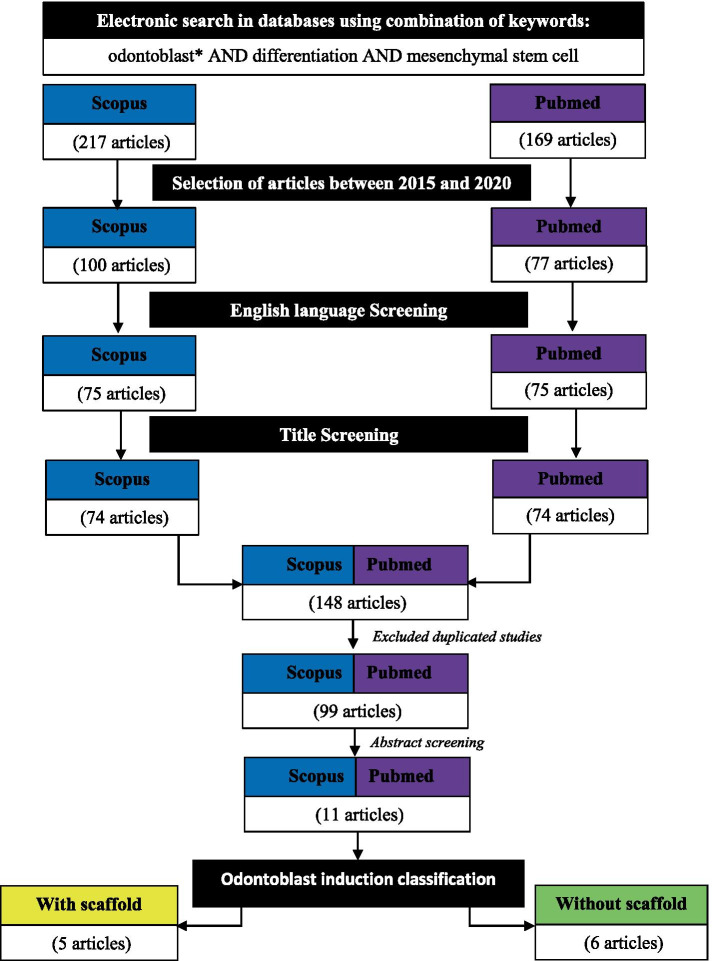
Flow chart of the article selection process from Pubmed and Scopus and databases

Two reviewers independently assessed the articles according to the defined inclusion and exclusion criteria. This procedure was performed to minimise bias while selecting the articles. At the end of the selection session, a joint discussion was conducted to achieve consensus when differences emerged during assessment. The primary searches that used the combination of keywords (Sect. 2.2) identified a total of 386 articles from both Scopus (217 articles) and PubMed (169 articles) combined. These articles were filtered to include publication date from 2015 to 2020, include research artlicle only and include English only, which resulted in only 148 articles selected. From this number, 99 duplicates were removed. After assessment of full study, 88 articles were excluded based on the criteria: these articles were not related to odontoblast differentiation, and some of them are in vivo studies. The flow chart of the selection process, which resulted in only 11 articles selected for review, is shown in Fig. 2.

### Study characteristics

All studies were published between 2015 to 2020 and reported on in vitro studies. The database search provided 11 articles related to stem cells, non-stem cells, odontoblastic differentiation approach and expression of odontoblast markers. Five out of 11 articles utilised scaffold for odontoblastic induction, while the rest were based on non-scaffold induction method. The results are measured by whether the initial expression of odontoblast markers is upregulated or enhanced after specific induction method was used. For the generated data, articles were classified into type of cells undergone odontoblastic differentiation, odontoblastic differentiation techniques using scaffold or non-scaffold. A summary of the studies is provided in Tables 1 and 2.

**Table 1:** Summary and classification of the articles by using non-scaffold to induce odontoblast

No	Author	Year	Cell type	Odontoblastic induction methods	Result	Conclusion
1	Hyun Jung Oh et al	2015	- MDPC-23 - ALCs - DPCs - C3H10T1/2 - HEK293	- Transfection of MDPC-23 cells with constructs encoding Cpne7 and Cpne7 siRNA	- Recombinant CPNE7 treated DPCs culture increased expression of DSPP mRNA and DSPP - Mineralized nodule formation was enhanced, and dentin/pulp-like tissue formation observed in DPCs	Cpne7 regulates odontoblast differentiation and dentin formation in vitro
2	Naoki Umemura et al	2016	- hDPSCs	- Analysis of CD44 expression of DPSC and effects of hyaluronan on the cell cycle - Measurement of BMP-2, BMP-4, DSPP and DMP-1 levels - Examination of DPSC cell signalling	- Number of CD44-expressing cells increased following treatment with HA - ALP proteins level increased in a concentration-dependent manner - Compared to control, the expression of DMP-1 and DSPP mRNA level increased in 7.7-fold and 6.7fold respectively in HA treated cells	- HA induces odontoblastic differentiation in DPSCs via CD44, but does not promote cell proliferation
3	Zhuo Chen et al	2016	- iMDP-3	- iMDP23 were induced with odontogenic medium - cells were transfected with Klf10 expression vector	- Klf10 upregulated the expression of DMP-11, DSPP and RUNX2 in iMDP-3 differentiation	- Klf10 promotes odontoblastic differentiation via the up-regulation of DMP1 and DSPP transcription
4	Jihua Chai et al	2019	- hDPSCs from third molar - Blood samples from healthy patients	- Effects of liquid PRF and PRP were assessed for cellular migration, proliferation and odontoblastic differentiation	- Liquid PRF treated cells showed significant increase in migration and greater ALP activity - When liquid PRF treated cells was cultured within inflammatory environment, the reduced regenerative potential was improved, facilitating hDPC regeneration	- Liquid PRF attenuated the inflammatory condition created by LPS and maintained a supportive regenerative ability for the stimulation of odontoblastic differentiation and reparative dentin in hDPCs
5	Lu Yan et al	2017	- Human healthy premolars and third molars	- The cells were cultured in odontoblastic induction medium containing various concentrations of high glucose	- Exposure to high glucose (25 and 50 mM) inhibited odontoblastic mineralization and reduced ALP activities - IGF-1 significantly reversed the effects of high glucose by restoring ALP activity and mineralization of DPCs	- IGF-1 restores the reduction of ALP activity and mineralization induced by high glucose. This indicates that IGF-1 attenuates the high glucose-induced inhibition of DPC proliferation, differentiation and mineralization

*MDPC-23* mouse dental papilla cell-23, *ALCs* Ameloblast-lineage cell, *HEK293* human embryonic kidney cells, *Cpne7* Copine 7, *DSPP* dentin sialophosphoprotein, *hDPSC* human dental pulp stem cells, *BMP* bone morphogenetic protein, *DMP* dentin matrix protein, *ALP* alkaline phosphatase, *HA* hyaluronan, *iMDP23* immortalized dental papilla mesenchymal cells, *Klf10* Kruppel like factor 10, *RUNX2* RUNX family transcription factor 2, *PRF* platelet-rich fibrin, *PRP* platelet-rich plasma, *IGF-1* insulin-like growth factor-1

**Table 2:** Summary and classification of the articles by using scaffold to induce odontoblast

No	Author	Year	Cell type	Odontoblastic induction methods	Result	Conclusion
1	Soares et al	2017	- Healthy human third molars	- hDPSCs were seeded on NF-PLLA scaffolds that mimic the nanofibrous architecture of extracellular matrix, and cultured with simvastatin and/or pro-inflammatory stimulator LPS	- Treating LPS + DPC/NF-PLLA constructs with simvastatin reverted negative effects of LPS on expression of odontoblastic markers, associated to the reduction in NFkBp65 phosphorylation and upregulation of PPARγ expression - The DPC/NF-PLLA constructs treated with LPS/simvastatin increased vessel-like structures, which corelated to increased VEGF expression in the DPSCs	- Combination of low dosage simvastatin and NF-PLLA scaffolds promotes dentin regeneration in inflamed dental pulp tissue
2	Yuanwei Chen et al	2015	- hUCMSCs	- TGC-CM was added to induce hUCMSCs into odontoblast-like cells - Preparation of hTDM by staining with HE and Masson’s trichrome - After the preparation of hTDM, hUCMSCs were differentiated under the odontogenic microenvironment provided by hTDM	- Induction of hUCMSCs with TGC-CM upregulate the expression of DSP and DMP-1 - hTDM was positive for DSPP and DMP-1 especially around dentin tubules, indicating that the dentin expressed DSPP and DMP-1	- hUCMSCs have an odontogenic differentiation potential to differentiate into odontoblast-like cells in an odontogenic microenvironment provided by TGC-CM and hTDM in vitro
3	Chunyang Huang	2020	- hDPSCs - hUCMSCs	- hDPCs and hUCMSCs were cultured in different concentrations of hydrogel to explore the more suitable concentrations for subsequent experiments	- hUCMSCs and hDPCs are viable and able to proliferate in 0.25% hydrogel scaffold - Compared with hUCMSCs-monoculture and hDPCs-monoculture, the co-culture groups exhibited more proliferative potential, alkaline phosphatase activity and mineralization nodule formation (*P* < 0.05)	- The co-culture of HUCMSCs and hDPSCs in hydrogel scaffold could regulate cell proliferation and differentiation within a certain range
4	Mohammad Chair Effendi et al	2015	- SHEDs	- SHED was cultured with MTA dose 2 mg and NMT dose 2 mg	- NMT was non-toxic towards SHED and increased the proliferation of SHED, and did not impede SHED viability especially on the second day compared to MTA - NMT could increase activity of ALP and DSPP compared to MTA on the SHED	- NMT could increase proliferation of SHED, increased ALP and DSPP activity in SHED and did not impede SHED viability
5	Tang, J. et al	2015	- MDPC-23 from foetal mouse first molar papillae	- Biocompatibility of type I collagen was evaluated in terms of initial cell number, ALP activity ALP activity, odontogenic gene expression and calcific deposition	- Cells cultured in type I collagen-modified substrate was induced to differentiate toward odontoblast lineage as demonstrated by upregulation of ALP activity on day 7, enhancement of ALP, BSP mRNA expression on day 7 and 10, and accelerated mineralization on day 9	- TS collagen accelerated early and late stage cellular differentiation as evidenced by enhancement of ALP activity and promotion of BSP, ALP, and OCN mRNA expression - Mineralization was dramatically accelerated in cells cultured on TS collagen
6.	Shunro Miyashita et al	2017	- hDPSC (first premolar and third molar, 14–28 years old) - hBMMSC - hAMC	- Odontoblastic differentiation in response to mechanical compression of three-dimensional scaffolds with dentinal tubule-like pores - Cell density of 4.0 × 105 cells/cm2; compression magnitude of 19.6 kPa; and loading time of 9 h were considered optimal conditions for differentiation	- hDPSCs cultured on 3D scaffolds significant increase expression of DSPP and enamelysin - Expression of DSPP and enamelysin in hBMMSCs and hAMCs with mechanical compression were similar to those in hDPSCs	- It may be possible to differentiate even the other tissue-derived hMSCs into odontoblasts by mechanical compression on membranes with pores like dentinal tubes

*hDPSC* human dental pulp stem cells, *NF-PLLA* nanofibrous poly (L-lactic acid), *LPS* lipopolysaccharide, *PPARγ* Peroxisome proliferator-activated receptor gamma, *DPC* dental pulp cell, *hUCMSC* human umbilical cord mesenchymal stem cell, *TGC-CM* tooth germ cell conditioned medium, *hTDM* human tooth dentin matrix, *HE* hematoxylin and eosin, *SHED* stem cells from human exfoliated deciduous teeth, *MTA* Mineral trioxide aggregate, *NMT* nanoparticle mineral trioxide, *ALP* alkaline phosphatase, *MDPC-23* mouse dental papilla cell-23, *BSP* bone sialoprotein, *OCN* osteocalcin, *TS* tilapia scale, *hBMMSC* human bone marrow derived mesenchymal stem cell, *hAMC* human amniotic mesenchymal cell

### Risk of Bias Assessment

In general, the studies included have a low risk of bias. Appropriate comparison groups and identical experimental conditions were included across all studies, preventing confounding elements from affecting the outcome of the studies. All studies have a low risk of selection, performance, attrition, detection, and reporting bias (Table 3).

**Table 3:** Risk of bias assessment for included studies

		[27]	[7]	[39]	[35]	[10]	[60]	[19]	[8]	[15]	[26]	[9]
Selection	Number across groups were matched	+	+	+	+	+	+	+	+	+	+	+
	Concealment of exposure allocation	NA	NA	NA	NA	NA	NA	NA	NA	NA	NA	NA
	Appropriate comparison group	+	+	+	+	+	+	+	+	+	+	+
	Absence of confounding factors	+	+	+	+	+	+	+	+	+	+	+
Performance	Identical experimental condition across groups	+	+	+	+	+	+	+	+	+	+	+
	Blinded outcome assessor	NA	NA	NA	NA	NA	NA	NA	NA	NA	NA	NA
Attrition	Outcome data were complete with no exclusion from analysis	+	+	+	+	+	+	+	+	+	+	+
Detection	Appropriate exposure assessment	+	+	+	+	+	+	+	+	+	+	+
	Appropriate outcome assessment	+	+	+	+	+	+	+	+	+	+	+
Reporting	All measured outcomes were reported	+	+	+	+	+	+	+	+	+	+	+

## Discussion

### Types of stem cells undergone odontoblastic differentiation

#### Dental pulp stem cells (DPSCs)

In all of the studies that are using DPSCs, most of the stem cells from were isolated from third molars pulp tissue, except for two studies conducted by Umemura et al. [7] and Huang et al. [8]. They did not specify the sample that they used to acquire the DPSCs. Shunro Miyashita et al. [9] and Yan et al. [10] on the other hand used both first premolar and third molar for their studies. There is no inflamed pulp tissue used in any of the studies, even though Alongi et al. [11] reported that it is an appropriate source for isolation of DPSCs. It has also been reported that stem cells from an exposed pulp are more prone to differentiate into osteoblastic cells rather than dentinogenic cells [12]. DPSCs, compared with bone marrow-derived mesenchymal stem cells (BMMSCs) and adipose-derived stem cells (ADSCs), needed longer time to become fully confluent after isolation. However, DPSCs exhibited a significantly higher viability compared to that of BMMSCs after two weeks cryopreservation, and showed a higher level of colony formation and proliferation rate and mineralization potential [13, 14]. The previous study has indicated that hDPSCs release many odontogenic markers such as alkaline phosphatase (ALP), type I collagen, osteocalcin (OCN), and dentin sialophosphoprotein (DSPP; [4]). Nevertheless, the factors that regulate and induce the odontoblastic differentiation of hDPSCs are complicated and still poorly understood.

#### Stem cells from human exfoliated deciduous teeth (SHED)

Out of eleven studies selected, only one study [15] conducted the experiment by using SHEDs. SHEDs are another type of stem cell, which are derived from extracted deciduous teeth and are considered as a non-invasive source of stem cells. These stem cells have an enhanced capacity for osteogenic regeneration and higher proliferation rate compared with DPSCs.

#### Bone marrow-derived mesenchymal stem cells (BMMSCs)

BMMSCs are another source that has been used extensively in regenerative procedures. Use of such cells with a dentine matrix scaffold was associated with differentiation of the stem cells into polarized odontoblast-like cells with penetrating processes into dentinal tubules [16]. Recent evidences have shown the uses of MSCs from non-odontogenic sources in tooth repair, highlighting the opportunities for use of non-dental cells in dental tissue regeneration and engineering [17]. However, as well as having a suitable cell source, appropriate morphogenic signals are required for odontoblast-like cell differentiation during dentine-pulp regeneration in an erupted tooth. If BMMSCs are to provide a suitable cell source for pulp regeneration, an alternative morphogenic signalling source to the odontogenic epithelium involved in tooth development will be required. One study included BMMSCs in odontoblastic differentiation potential [9].

#### Wharton’s jelly-derived mesenchymal stem cells (WJMSCs)

WJMSCs have a high proliferative capacity. They do not turn into teratogenic or carcinogenic cells in case of transplantation [18]. Umbilical cords, which are the sources for WJMSCs, are available in large volumes without invasive harvesting procedures. Multiple studies have shown that WJMSCs have the capacity for differentiation into odontoblast-like cells and deposition of hard tissue. Notably, these cells are considered safe as they are protected from viral infections by the placenta, which has a significant clinical importance [19]. Out of eleven studies selected, two studies used WJMSCs as one of their cell type [8, 19].

#### Human amniotic fluid mesenchymal stem cells (hARMSCs)

One of the studies used hAFMSCs in order to compare its expression of DSPP and enamelysin to that of hDPSCs and hBMMSCs [9]. Adult stem cells are limited in their differentiation potential and even after reprogramming, they maintain epigenetic modifications which may restrict their application. Fetal stem cells may overcome these limitations. It is established that umbilical cord and placenta are significant alternatives. Other than that, the amniotic fluid is an appealing cellular reservoir during gestation. Ethical concerns associated with its isolation is minimal [20], as it can be collected safely during second trimester routine amniocentesis, third trimester amnioreduction or caesarean section (end of gestation). Amniotic fluid mesenchymal stem cells (AFMSCs) have great potential in therapeutic applications and several methods of isolation and expansion have been described. Their ability to repair muscle, cartilage and bone defects have been tested in established animal models [21–24].

### Types of non-stem cells undergone odontoblastic differentiation

#### Mouse dental papilla cell-23

The mouse dental papilla cell-23 (MDPC-23) cell line was developed as a spontaneously immortalized cell line derived from fetal mouse first molar papillae cells and cloned specifically to have high ALP activity, the ability to form multilayered nodules and a cell doubling time of less than 24 h. MDPC-23 cell line makes transcription products for DSP, type-I collagen, ALP, OPN and osteocalcin (OCN) [25]. Out of eleven studies selected, two studies used MDPC-23 as their choice of cell type [26, 27].

#### Immortalized mouse dental papilla mesenchymal cells

In this study, immortalized mouse dental papilla mesenchymal cell lines were generated from the first mouse mandibular molars at postnatal day 3 using pSV40. The data from previous study suggested that iMDP-3 is one of the cell lines that displayed a high proliferation rate but retained the genotypic and phenotypic characteristics similar to primary cells as determined by expression of tooth-specific markers and demonstrated the ability to differentiate and form mineralized nodules [28]. iMDP-3 cells also had high transfection efficiency as well as were inducible and responded to BMP2 stimulation.

### Odontoblastic differentiation using non-scaffold

#### Insulin-like growth factor-1 (IGF-1) promotes odontoblastic differentiation

Previous study done by Joseph et al.found that secretory ameloblasts, secretory odontoblasts and mature ameloblasts express high levels of IGF-1 in the development of the rat incisor [29]. It has also been demonstrated that IGF-1 promotes hDPSCs proliferation and osteogenic differentiation by increasing the expression of differentiation markers through the mammalian target of rapamycin (mTOR) signalling pathway [30]. Results by Yan et al.showed that high glucose (GLU), specifically 25 mM GLU significantly decreased OCN, ON, OPN, DSP and DMP-1 expression in hDPSCs during differentiation. However, IGF-1 significantly reversed the effects of high GLU. IGF-1 restored ALP activity and promoted odontoblastic differentiation by increasing the expression levels of mineralization-related proteins as mentioned above [10].

#### Liquid platelet-rich fibrin and platelet-rich plasma

Platelet concentrates are a concentration of autologous growth factors derived from peripheral blood which are reported to have regenerative potential. Platelet-rich plasma (PRP) has been shown to release the majority of its growth factor content within an early healing period (within 8 h) [31, 32]. Platelet-rich fibrin (PRF) was developed because it does not use anticoagulants, unlike PRP. Furthermore release of growth factors from the fibrin clots of PRF sustains a longer and more gradual release of growth factors over time which is ideal for tissue repair. It has been previously reported that the combination of PRP and PRF with hDPSCs improved pulp regeneration in a canine tooth model [33, 34]. In 2019, Chai et al. did a study that compared the cellular regenerative activity of hDPSCs when cultured with PRF or PRP [35]. It is reported that PRP increased the expressions of DSPP and DMP-1.

#### Hyaluronan induces odontoblastic differentiation of DPSC

Odontoblasts, especially those in the root ends of immature teeth, express CD44, which is strongly expressed by cells undergoing mineralization, such as ameloblasts, odontoblasts and osteoblasts in calcifying areas. CD44 functions as an adhesion molecule and is broadly distributed type I transmembrane glycoprotein receptor for the glycosaminoglycan hyaluronan (HA). When DPSCs were cultured in HA for 24 h, BMP-2 and BMP-4 mRNA levels underwent no significant changes, while DSPP and DMP-1 mRNA levels were markedly increased. The DMP-1 mRNA level increased 7.7-fold, while that of DSPP increased 6.7-fold. HA also increased DMP-1 and DSPP protein levels. These results suggest that HA stimulated DPSCs toward odontoblastic differentiation via CD44 signalling even though HA does not promote cellular proliferation [7].

#### Transfection of MDPC-23 cells with CPNE7 and CPNE7 siRNA

CPNE7 is the protein identified as one of the dental epithelium-derived factors present in the conditioned medium of pre-ameloblasts (PA-CM). In one of the studies selected, Oh et al. [27] investigated biological function and mechanisms of CPNE7 in regulation of dental and non-dental mesenchymal cell differentiation into odontoblasts via epithelial-mesenchymal interaction. Based on previous reports, CPNE7 mRNA and protein increased during odontoblastic differentiation, and stimulation of CPNE7 promotes expression of odontoblast-related genes, including DSPP, OCN and ALP [36]. It is observed that the expression of DSPP was upregulated by CPNE7 overexpression or rCPNE7 treatment. rCPNE7 promoted mineralized nodule formation in vitro. Endogenous CPNE7 was expressed in MDPC-23 cells from the beginning of the culture even without PA-CM induction. It is well known that MDPC-23 cells can differentiate into odontoblasts without dental epithelial induction because the cells were already induced by underlying inner enamel epithelium so that they could express CPNE7. Endogenous CPNE7 expression was enhanced by co-culture with ALCs or rCPNE7 treatment. In short, CPNE7 induced differentiation of odontoblast-like cells from mesenchymal cells of dental or non-dental origin.

#### Transfection of iMDPC-3 with Klf10 expression vector

Klf10 is a part of the Kruppel-like family of transcription factors and was identified in normal human foetal osteoblasts (hFOB) after TGFβ treatment, by using differential display polymerase chain reaction (PCR) method [37]. Klf4 promotes the differentiation of odontoblasts via the up-regulation of DMP-1 [38]. In addition, Klf10 plays an important role in regulating osteoblast differentiation. Both osteoblasts and odontoblasts are derived from mesenchymal cells and mechanisms of osteogenesis and dentinogenesis resemble each other in critical steps. A study was done to examine Klf10 expression in an iMDP-3 [39]. mRNA level of DMP-1, DSPP and Runx^2^ increased in the Klf10 overexpression group, which resulted in induced cell differentiation into odontoblast and mineralization in iMDP-3.

### Odontoblastic differentiation using scaffold

#### Cells co-culture in hydrogel-cell complex

Due to the shortage of dental pulp cells, many researchers co-cultured stem cells with dental pulp cells to overcome the lack of cell source and achieve pulp regeneration. Jia et al. (2017) in their past study managed to establish a co-culture system (hDPSCs and hUCMSCs cultured together) where the cell proliferation was increased and osteogenic genes expression was enhanced [40]. Based on this study, Huang et al. conducted another study where hDPSCs induced by BMP-2 were divided into three groups, hDPSCs group, hUCMSCs group and co-culture group (hDPSCs and hUCMSCs were cultured in 1:1 ratio). This mode of cell culture was closer to the environment of cell growth in vivo. The results showed that hDPCs and hUCMSCs could grow and proliferate in hydrogel scaffolds [8].

#### Silicon membranes with mechanical forces

Several in vitro studies have shown that mechanical stimulation induces the differentiation of MSCs into osteoblasts [41–43] and chondrocytes [44, 45]. Promotion of the differentiation of hDPSCs into odontoblasts by mechanical forces in vitro has been demonstrated by mRNA expression of the odontoblastic markers DSPP and DMP-1 [46]. Shunro et al. [9] conducted a study where they determined the optimal conditions for the induction of hDPSCs into odontoblastic differentiation in response to mechanical compression of three dimensional (3D) scaffolds with dentinal tubule-like pores. Their results established that the optimal conditions that are able to induce odontoblastic differentiation of hDPSCs are: cell density 4.0 × 10^5^ cells/cm^2^, compression magnitude of 19.6 kPa and loading time of 9 h. This is evaluated by specific marker expression and morphological features of odontoblasts. hDPSCs without mechanical compression showed little odontoblastic differentiation, indicating the vital role of mechanical compression for the odontoblastic differentiation of hDPSCs. It is proposed that odontogenic differentiation of hDPSCs by mechanical compression is done via the MAPK signalling pathway [47].

#### Human tooth dentin matrix (hTDM)

Past studies have shown that tooth germs of Sprague–Dawley for secretome (TGC-CM) provides a microenvironment equipped with regulating factors for tooth morphogenesis which enhanced odontogenic differentiation of dental as well as non-dental stem cells [4, 5]. There are also various scaffolds for tooth regeneration such as polyglycolic acid and collagen [48]. Human tooth dentin matrix (hTDM) is a scaffold that maintains major structure of dentin tubules, while it also expresses DSP and DMP-1 which are critical in dentogenesis. In short hTDM not only serves as a scaffold, it also provides an odontoblastic microenvironment for stem cells [49, 50]. Chen et al. [19] in their study demonstrated that hUCMSCs can be differentiated into odontoblast-like cells by hTDM in vitro, and that the proliferation rate of hUCMSCs was not altered after combining with hTDM. The in vivo part of the study showed that newly formed calcifications were observed after hTDM-hUCMSCs composites were implanted subcutaneously into nude mice for two months.

#### Nanoparticle Mineral Trioxide (NMT)

Mineral trioxide aggregate (MTA) is a material commonly used in endodontics. It is assumed that the effectiveness of MTA can be increased by modifying the size of MTA particles to be nanoparticles, specifically nanoparticle mineral trioxide (NMT). Effendi et al. examined whether modification of MTA to NMT could increase and stimulate the rate of proliferation and differentiation of SHEDs to odontoblasts by quantifying differentiation and maturation markers [15]. MTA particle size was modified using a high energy milling machine (HEM). The results indicated that NMT can increase SHEDs proliferation, and was found not toxic towards SHEDs. Both ALP and DSPP activities were found increased as well.

#### Type-I collagen derived from tilapia scale

The main component of fish scale is hydroxyapatite and type-I collagen, which are similar to that of human dentin and bone. Previous studies have characterized the properties of type-I collagen derived from fish scale [51–53], and reported the potential application of as biomaterials in tissue engineering [54–56]. Based on this knowledge, Tang and Saito [26] investigated the growth, differentiation, mineralization and morphology of MDPC-23 when cultured on tilapia scale collagen (TS collagen). The cell morphology photographs and number suggested that triple helical TS collagen acted as a sticky cue to attract more cells to anchor to the culture plate. This property has many benefits in dentin regeneration, since it is preferred that the material be able to recruit odontoblast and initiate earlier cellular differentiation. TS collagen accelerated early and late stage cellular differentiation as evidenced by enhancement of ALP activity and promotion of BSP, ALP and OCN mRNA expression. Mineralization was significantly accelerated too in cells cultured on TS collagen.

#### Simvastatin and nanofibrous poly(L-lactic acid) (NF-PLLA) scaffolds

It has been shown that NF-PLLA scaffolds include hDPSCs to differentiate into highly secretive odontoblast-like cells in vitro [3, 57]. The cells also maintained their differentiated phenotype and formed hard tissue after 8 weeks of subcutaneous implantation in nude mice. Simvastatin has emerged as a promoting agent for dentin regeneration due to its pleiotropic effects, including its ability to decreased inflammation, improve endothelial function and enhance mineralized tissue deposition by osteoblasts/odontoblast precursors [58, 59]. In one of the studies chosen, Soares et al.investigated the anti-inflammatory, odontogenic and pro-angiogenic effects of integrating simvastatin and NF-PLLA scaffolds on hDPSCs. Treating lipopolysaccharide (LPS) with DPC/NF-PLLA (LPS + DPC/NF-PLLA) constructs with simvastatin reverted the negative effects of LPS on expression of odontoblastic markers. These constructs also led to increase in vessel-like structures, which is related to VEGF expression in both DPSCs and endothelial cells [60].

## Conclusion

There are various materials that we can use to induce odontoblastic differentiation. One of the best methods is to combine the usage of scaffolds and growth factors. Growth factors from conditioned medium provide microenvironment equipped with regulating factors for tooth morphogenesis which enhanced odontogenic differentiation of stem cells. More studies need to be conducted to elucidate about the odontoblastic differentiation mechanism and how to enhance it.

## Data Availability

The datasets used and/or analysed during the current study are available from the corresponding author on reasonable request.

## Abbreviations

RE: Regenerative endodontics
MSC: Mesenchymal stem cell
DPSC: Dental pulp stem cells
SHED: Stem cells from human exfoliated deciduous teeth
SCAP: Stem cells from apical papilla
DFSC: Dental follicle stem cells
GMSCS: Gingival mesenchymal stem cells
PDLSC: Periodontal ligament stem cells
EMC: Ectomesenchymal cells
DMP: Dentin matrix proteins
TGC-CM: Tooth germ cell-conditioned medium
BMMSC: Bone marrow-derived mesenchymal stem cells
ADSC: Adipose-derived stem cells
ALP: Alkaline phosphatase
OCN: Osteocalcin
DSPP: Dentin sialophosphoprotein
AFMSC: Amniotic fluid mesenchymal stem cells
MDPC-23: Mouse dental papilla cell-23
PRP: Platelet-rich plasma
PRF: Platelet-rich fibrin
PA-CM: Conditioned medium of pre-ameloblasts
TGC-CM: Tooth germs of Sprague–Dawley for secretome
hTDM: Human tooth dentin matrix
MTA: Mineral trioxide aggregate
NMT: Nanoparticle mineral trioxide
HEM: Energy milling machine
NF-PLLA: Nanofibrous poly(L-lactic acid)
